# Response of *Brassica napus* to *Plasmodiophora brassicae* Involves Salicylic Acid-Mediated Immunity: An RNA-Seq-Based Study

**DOI:** 10.3389/fpls.2020.01025

**Published:** 2020-07-09

**Authors:** Leonardo Galindo-González, Victor Manolii, Sheau-Fang Hwang, Stephen E. Strelkov

**Affiliations:** Department of Agricultural, Food & Nutritional Science, University of Alberta, Edmonton, AB, Canada

**Keywords:** *Brassica napus*, *Plasmodiophora brassicae*, clubroot, RNA-seq, salicylic acid, nematodes, immunity

## Abstract

Clubroot, caused by the obligate parasite *Plasmodiophora brassicae*, is an important disease of the Brassicaceae and poses a significant threat to the $26.7 billion canola/oilseed rape (*Brassica napus*) industry in western Canada. While clubroot is managed most effectively by planting resistant host varieties, new pathotypes of *P. brassicae* have emerged recently that can overcome this resistance. Whole genome analyses provide both a toolbox and a systemic view of molecular mechanisms in host-pathogen interactions, which can be used to design new breeding strategies to increase *P. brassicae* resistance. We used RNA-seq to evaluate differential gene expression at 7, 14 and 21 days after inoculation (dai) of two *B. napus* genotypes with differential responses to *P. brassicae* pathotype 5X. Gall development was evident at 14 dai in the susceptible genotype (the oilseed rape ‘Brutor’), while gall development in the resistant genotype (the rutabaga (*B. napus*) ‘Laurentian’) was limited and not visible until 21 dai. Immune responses were better sustained through the time-course in ‘Laurentian’, and numerous genes from immune-related functional categories were associated with salicylic acid (SA)-mediated responses. Jasmonic acid (JA)-mediated responses seemed to be mostly inhibited, especially in the resistant genotype. The upregulation of standard defense-related proteins, like chitinases and thaumatins, was evident in ‘Laurentian’. The enrichment, in both host genotypes, of functional categories for syncytium formation and response to nematodes indicated that cell enlargement during *P. brassicae* infection, and the metabolic processes therein, share similarities with the response to infection by nematodes that produce similar anatomical symptoms. An analysis of shared genes between the two genotypes at different time-points, confirmed that the nematode-like responses occurred earlier for ‘Brutor’, along with cell metabolism and growth changes. Additionally, the susceptible cultivar turned off defense mechanisms earlier than ‘Laurentian’. Collectively, this study showed the importance of SA in triggering immune responses and suggested some key resistance and susceptibility factors that can be used in future studies for resistance breeding through gene-editing approaches.

## Introduction

Clubroot, caused by the obligate parasite *Plasmodiophora brassicae* Wor., is a soilborne disease of the Brassicaceae and represents a major threat to canola/oilseed rape (*Brassica napus* L.) production. In Canada, where the canola crop contributes $26.7 million annually to the national economy (Canola Council of Canada – Industry overview, 2017), clubroot has been spreading rapidly through the Prairies and has become a significant challenge for many farmers (Strelkov et al., 2015; Strelkov et al., 2019). Disease development is associated with the production of large numbers of pathogen resting spores, which can persist in the soil for many years and serve as inoculum for the infection of subsequent crops (Howard et al., 2010; Hwang et al., 2012). Strategies for the management of clubroot include crop rotation, sanitization of field equipment, and the application of fungicides and soil amendments (Donald and Porter, 2009). Unfortunately, many of these strategies are not practical or cost-effective (Hwang et al., 2014), and as such farmers have relied mostly on the planting of clubroot resistant (CR) cultivars. In recent years, however, new pathotypes of *P. brassicae*, capable of overcoming the resistance in most CR canola cultivars, have been identified with increasing frequency (Strelkov et al., 2016b; Strelkov et al., 2018). The emergence of these new pathotypes highlights the need for additional sources of resistance and a better understanding of the host response to infection.

The clubroot pathogen invades plants through the roots, causing a primary infection in the root hairs and then a secondary infection of the cortical tissues (Kageyama and Asano, 2009; Hwang et al., 2012). The secondary plasmodia that develop in the cortex trigger host regulation of growth related hormones (auxins, brassinosteroids, cytokinins), resulting in hypertrophy and hyperplasia that lead to gall formation (Kageyama and Asano, 2009; Ludwig-Müller et al., 2009; Schuller et al., 2014; Ludwig-Müller et al., 2017). Cell enlargement has a two-fold benefit for *P. brassicae*, housing the enlarged plasmodia which divide concomitantly with the host cells, and providing increased nutrients for pathogen growth and reproduction (Ludwig-Müller et al., 2009; Jahn et al., 2013; Schuller et al., 2014). Therefore, early studies on the molecular basis of clubroot were focused on hormonal control, but now have expanded further into analyzing whole genome defense responses.

Genetic and molecular studies to increase resistance to *P. brassicae* have traditionally focused on finding resistance genes or resistance loci that can be introgressed into cultivars by classical breeding approaches using diverse marker techniques. Two such genes (*Crr1* and *Crr2*) were first identified in *Brassica rapa* L. in an F_2_ segregating population, showing that they acted together to confer resistance (Suwabe et al., 2003). *Crr1* was subsequently characterized as consisting of two genes, *Crr1a* corresponding to a TIR-NBS-LRR protein and a second locus (*Crr1b*) that was not characterized, but which seems to have a minor effect on resistance (Hatakeyama et al., 2013). Another resistance gene (*CRa*) was originally introgressed from turnip (*B. rapa* ssp. *rapifera*) and mapped by linkage analysis in Chinese cabbage (*B. rapa* ssp. *pekinensis*) (Matsumoto et al., 1998), and then fully characterized as a TIR-NBS-LRR protein (Ueno et al., 2012). The *Rcr1* locus, which was linked to clubroot resistance in *B. rapa* ssp. *chinensis*, was associated with differential expression of defense mechanisms in genotypes with or without the allele (Chu et al., 2014). The region encompassing this marker includes at least 35 genes, of which two (*R-*genes) have the larger number of polymorphisms associated with differences in resistance (Yu et al., 2016)

Mapping in *B. napus* has shown different sources of resistance located on diverse chromosome locations depending on the specific genotype/cultivar-isolate interaction. Such resistance can come from a major gene (Manzanares-Dauleux et al., 2000; Hasan and Rahman, 2016; Zhang et al., 2016a), or from several QTLs (Jung, 2008; Li et al., 2016) that do not necessarily match resistance genes found in progenitor genomes or in other genomes from the family. This fact is probably related to genetic rearrangements which are common during polyploidization events (Szadkowski et al., 2011; Xu et al., 2012). For example, in *Brassica oleracea* L., some QTLs were found to co-localize with previously identified CR loci for that species, but are not analogous to CR loci from *B. rapa* (Lee et al., 2016). In *Arabidopsis thaliana* (L.) Heynh, several QTLs related to resistance do not map to previously characterized monogenic resistance in a different genotype (Jubault et al., 2008), and QTL regions encompass *R-*genes, as well as genes related to the cell wall, antimicrobial compounds, the oxidative burst, auxin metabolism, cell expansion and root architecture. These data support a mix of quantitative and qualitative resistance that is highly dependent on the host-pathogen interaction, and highlights the need for exploring key molecular regulatory mechanisms beyond *R* ** **-genes.

Transcriptome studies in the clubroot pathosystem have allowed the generation of a molecular landscape in susceptible and resistant interactions, which can serve as a scaffold for understanding the genomic basis of resistance. Studies analyzing host transcriptomic changes first used microarrays, but later switched to RNA-seq analysis, which allows discovery of novel transcripts and has a larger dynamic range for transcript level detection. Microarray analysis of the clubroot susceptible *A. thaliana* ecotype Col-0 challenged with *P. brassicae* showed transcriptional changes in cell growth, carbohydrate and defense mechanisms, and modulation of genes related to auxin, cytokinin and brassinosteroid metabolism (Siemens et al., 2006; Agarwal et al., 2011; Schuller et al., 2014). An alternate approach using microarrays was undertaken by using *A. thaliana* Bur-0, which showed partially resistant and susceptible reactions, respectively, to two different *P. brassicae* isolates (Jubault et al., 2013). This study indicated that the partially resistant interaction delays pathogen control of host metabolism and induces stronger or earlier defense responses.

The incremental shift towards use of RNA-seq has validated some of the results from microarrays, but also increased the level of information in genome-scale studies. One of the early studies with RNA-seq used *B. rapa* ssp. *chinensis* lines segregating for the clubroot resistance gene *Rcr1* (Chu et al., 2014). Resistant lines carrying the *R* **-gene upregulated jasmonate and ethylene metabolism, reinforced cell walls through callose deposition, and used indole-containing metabolites as defense mechanisms. More recent studies with *B. rapa* ssp. *pekinensis* highlight how resistant interactions involve receptors of pathogen recognition, pathogenesis-related proteins, salicylic acid signalling and secondary metabolism as key elements of defense (Chen et al., 2016a; Jia et al., 2017). In *Arabidopsis*, responses at 24 and 48 h after inoculation (period of primary infection by *P. brassicae*) of the susceptible ecotype Col-0 included secondary metabolite synthesis (flavonoids), lignification, hormone signalling (auxins and cytokinins) and receptor-like kinase activation (Zhao et al., 2017). Surveys of the same interaction at 17, 20 and 24 days post-inoculation (dpi) in shoots and roots showed downregulation of primary metabolism (photosynthesis, carbohydrate metabolism) and cell wall modification, while an upregulation of secondary metabolism-related genes (glucosinolates, camalexin, phytoalexins) was detected mainly in shoots (Irani et al., 2018). Roots showed features of disease development through upregulation of cell wall modification enzymes and auxin response genes, resulting in cell growth, division and expansion.

As RNA-seq becomes an important tool in understanding the molecular mechanisms of defense against *P. brassicae*, specific interactions between particular pathotypes and host genotypes can now be studied using this technology. Addressing specific pathotype-host genotype interactions is necessary to dissect constitutive and induced defenses and how these mechanisms may be altered when new interactions arise. Furthermore, a focus on understanding the responses of *B. napus* to specific pathotypes would enable a direct assessment of important genes in this host, rather than looking for homologous genes in close relatives. Selecting candidate genes in *B. napus* for functional validation, or alteration of resistance through gene editing, can shorten the time required for the development of new genotypes/cultivars with increased host resistance.

In the current study, we followed disease progression through a time-course in two *B. napus* genotypes having a contrasting interaction with pathotype 5X of *P. brassicae*, as classified on the Canadian Clubroot Differential set (Strelkov et al., 2018). This pathotype can overcome the resistance in most CR canola cultivars and is one of numerous novel pathotypes of the clubroot pathogen identified in western Canada in recent years (Strelkov et al., 2016a). Our study shows that the host (especially the moderately resistant genotype) relies heavily on salicylic acid-mediated immunity and that several defense mechanisms mimic defense mechanisms of plants when faced with infection by nematodes. Our research presents a complete description of the molecular responses in this specific interaction, outlining previously reported key defense genes, and shedding light on candidate genes that behave as defense and susceptibility factors.

## Materials and Methods

### Plant Material, Inoculations and Harvesting

Seeds of the rutabaga (*B. napus* subsp. *napobrassica* ‘Laurentian’) and the oilseed rape (*B. napus* var. *napus* ‘Brutor’) were germinated on moistened filter paper in Petri dishes for 1 week prior to inoculation. ‘Laurentian’ is characterized as resistant (index of disease, ID <50%) to pathotype 5X, while ‘Brutor’ is susceptible (ID >50%) to this pathotype (Strelkov et al., 2018).

Inoculum of pathotype 5X was prepared from galls collected initially from the canola (*B. napus* var. *napus*) ‘L135C' near Westlock, Alberta, Canada, in 2013 (Strelkov et al., 2016a). One hundred grams of galled root tissue were ground in 1 L of sterile distilled (sd)-water in a blender and filtered through eight layers of cheesecloth. The resting spore concentration in the filtrate was estimated using a haemocytometer and adjusted to 1 × 10^7^ spores/ml with sd-water.

One-week old seedlings used as controls were transferred directly to pots filled with water-saturated Sunshine LA4 potting mix (SunGro Horticulture, Vancouver, BC, Canada), while plants used for inoculation were placed in a Petri dish with the inoculum suspension for 15 s before transferring them to the pots. An additional 1 ml of the spore inoculum was added directly to the potting mix for the inoculated plants to ensure strong disease pressure.

The plants were placed in insect cages (47.5 cm × 47.5 cm × 93.0 cm) to avoid potential arthropod infestations that might interfere with the results, and maintained in a greenhouse under long day conditions (16 h) and an average temperature of 22°C. Six biological replicates were collected at 7, 14 and 21 days after inoculation (dai) for each of the control vs. inoculated treatments and for both genotypes studied. Biological replicates comprised 12 plants, which were pooled at the time of harvest for each time-point and treatment. Five replicates were harvested for downstream expression analysis and individual plants from the last replicate were used to collect samples for microscopy. The collected roots were washed thoroughly under running water, dried on a paper towel, placed in 50 ml Falcon tubes (Fisher Scientific, Hampton, NH, U.S.), and transferred immediately to liquid nitrogen. Root sections (0.5-cm long, up to 4 cm from the root base) were collected for microscopy analysis as described below.

### Microscopy

For the microscopy analysis, root tip sections (0.5 cm) were placed in fixative solution (10% neutral buffered formalin) for a minimum of 2 days. Samples were then placed in plastic cassettes and dehydrated in a 50–70–90–100% ethanol series in a Leica TP1020 tissue processor (Leica, Nussloch, Germany). Tissues were embedded in paraffin blocks using a TISSUE TEK II embedding center (Sakura, Torrance, CA, USA). Eight to 12 µm sections were obtained with a RM2125 microtome (Leica, Nussloch, Germany), and dried on microscope slides overnight at 37°C.

Staining was performed with Hematoxylin–Eosin according to the following procedure: slides were dewaxed in toluene for 5 min twice and then washed with 100% ethanol twice for 2-min, followed by a 2-min wash in each of three ethanol solutions (90, 70, 50%). The slides were washed in distilled water for 1–2 min, Hematoxylin Gill III solution (Leica, Nussloch, Germany) was added for 2 min, and then rinsed with distilled water. Next, the slides were placed under running tap water for 15 min, washed for 2 min with 70% ethanol, and Eosin (Leica) was added for exactly 30 s. The Eosin was washed with 100% ethanol twice for 2 min, followed by two cleaning steps using toluene for 2 min. DPX mounting medium (EMS, Hatfield, PA. U.S.A.) was added to the slides to fix the preparations and inhibit stain fading, and the sections were covered with a coverslip and incubated at 37°C overnight. The stained sections were examined under an Optika B-290TB light microscope (Ponteranica, Italy), and images were captured with the Optika Vision Lite v2.13 digital USB camera system.

### RNA Extraction

Frozen roots were placed in 15 ml Falcon tubes and ground using 5–7 mm-diameter steel beads at 1,100 to 1,200 rpm (3–4 rounds of 1 min each) in a Genogrinder (SPEX SamplePrep, Metuchem, NJ, U.S.A), followed by additional grinding in a mortar with a pestle in the presence of liquid nitrogen. Total RNA was extracted with a combination of TRIzol reagent (Thermo Fisher Scientific, Waltham, MA, U.S.A.) and the RNeasy kit (QIAGEN, Venlo, Netherlands) to increase the quantity and purity of the RNA. DNase treatment was performed on-column with the RNase-Free DNase set (QIAGEN, Venlo, Netherlands). The quantity of RNA was assessed with a Nanodrop 2000c spectrophotometer (Thermo Fisher Scientific, Waltham, MA, U.S.A.) and quality was checked in a 2200 TapeStation (Agilent, Santa Clara, CA, U.S.A.).

### RNA-Seq

Three micrograms of RNA per pooled sample corresponding to each biological replicate (see above), with RNA Integrity Numbers (RIN) > 9, were sent for sequencing at Oklahoma State Genomics (Stillwater, OK, U.S.A.). Libraries were prepared with a TruSeq Stranded mRNA library preparation kit for 48 samples (Illumina, San Diego, CA, U.S.A) and samples were sequenced using TG Nextseq 500/550 High Output Kit v2 (75 cycles), in a NextSeq500 equipment (Illumina, San Diego, CA, U.S.A). The raw data reads were filtered using the bcl2fastq conversion software provided by Illumina. This software uses read indexes to assign each read to the corresponding sample and then removes any adapters, barcodes, or primers using a trimmomatic-like algorithm. Any reads with an average Q-score <30 were removed and trimming of read ends was performed for bins of 15 bp that had a Q-score <30. Sequencing reads were deposited in the NCBI Sequence Read Archive (SRA) under accession number: PRJNA597078.

The reads from all treatments were mapped to the *B. napus* reference genome (AST_PRJEB5043_v1) using the genome and gene annotation deposited in Ensembl (Chalhoub et al., 2014; Ensembl, 2018; Kersey et al., 2018). Genome indexes were generated with Bowtie2 v2.3.3.1 (Langmead and Salzberg, 2012) and transcriptome indexes with TopHat v2.1.1 (Trapnell et al., 2009). Reads mapped with TopHat were used along gene models (obtained from the Ensembl website) as input for Cufflinks v.2.2.1 to generate all potential transcripts in each sample. Cufflinks was run using the GTF-guide option, multi-read correction and fragment bias correction (multi-read correction and fragment bias correction were also used in the subprograms downstream). GTF files generated from all treatments and replicates were used to generate a consensus merged transcript gtf file with Cuffmerge. This consensus file was employed as a reference for re-mapping reads for quantification. Cuffquant was run with the merged gtf file to create ready-to-use files with quantified transcripts, to perform the necessary comparisons of differential expression among treatments. Cuffdiff was used to compare cuffquant files, contrasting controls with inoculated plants at each time-point and for each genotype separately. Expression levels were quantified as Fragments Per Kilobase of transcript per Million mapped reads (FPKM) and significant differential expression for each evaluated transcript was assessed after multiple comparison testing with the Benjamini-Hochberg correction (Benjamini and Hochberg, 1995).

The number of mapped reads from all treatments was compared using a one way ANOVA implemented in R. FPKM values of all genes from treated and inoculated replicates were log-transformed to build PCA plots using the R-package ggfortify (https://github.com/sinhrks/ggfortify) (Son et al., 2018).

### cDNA Synthesis and Quantitative Reverse Transcription PCR (qRT-PCR)

Five hundred nanograms of RNA per sample were subjected to DNAse treatment using RNase-free-DNase I (Thermo Fisher Scientific, Waltham, MA, U.S.A.). cDNA was synthesized using the RevertAid H Minus Reverse transcriptase with oligo dT (18), following manufacturers' specifications (Thermo Fisher Scientific). To test for residual DNA contamination, a 1:100 dilution of cDNA was used to perform PCR with primers corresponding to a constitutively expressed Clathrin Adaptor Complex (CAC) gene (**Supplementary Table S1**), producing 125 bp and 288 bp bands on cDNA and DNA, respectively. PCR analysis was conducted with 1× Buffer + KCl, 2.5 mM MgCl_2_, 0.2 mM of each dNTP, 0.2 µM or each primer, and 1 unit of high fidelity Taq polymerase. Cycling conditions were 94°C for 3 min, followed by 35 cycles of 94°C for 30 s, 60°C for 30 s, and 72°C for 1 min, finishing with an extension step of 72°C for 10 min, and a 4°C step until samples were recovered from the thermocycler.

To validate the differential expression detected by RNA-seq analysis, 15 primer pairs from genes showing significant differential expression in at least one time-point or host genotype were evaluated for efficiency (range of 90 to 110%) using a 5-point dilution series (1:4 serial dilutions) of a mix of all cDNA samples. Ten target primers within the expected efficiency range were used for final RNA-seq validation (**Supplementary Table S1**). Five reference gene primers with valid efficiencies were tested for stability across all samples using BestKeeper (Pfaffl et al., 2004), and the three genes showing the best correlation and lower standard deviation were chosen for relative quantification (**Supplementary Table S1**). Primers were designed using Primer3 (NIH, 2012; Untergasser et al., 2012) with the following parameters: Na^+^ concentration 50 mM, Mg^++^ concentration 3 mM, dNTPs concentration 0.8 mM, oligo length: min 18, opt 22, max 30 bp, melting temperature: min 60, opt 62, max 64°C, GC content: min 35, opt 50, max 65%, amplicon length: min 70, opt 100, max 250 bp.

Relative quantification of expression was performed using the 2^−ΔΔ^CT method (Livak and Schmittgen, 2001). All qRT-PCR experiments were performed with four biological replicates and three technical replicates per biological replicate. The experiments were run on a ViiA7 Real-Time PCR system (Applied Biosystems-Life Technologies, Carslbad, CA, USA). Reactions were performed in 10 µl with 5 µl of an in-house made SYBR-green reagent mix, 2.5 µl of the pair of mixed primers (3.2 µM), and 2.5 µl of a 1:100 dilution of cDNA. Cycling conditions were 95°C for 2 min followed by 40 cycles of 95°C for 30 s and 60°C for 1 min. A melting curve stage was added to verify product specificity: 95°C for 15 s, 60°C for 1 min, and 95°C for 15 s.

Log_2_-fold change values from the qRT-PCR analysis were compared with the RNA-seq values of the respective genes to validate the RNA-seq data. Correlation coefficients were calculated using the CORREL function from Microsoft Excel (Microsoft, Redmon, WA, U.S.A.) for each day and host genotype using the 10 target genes.

### Bioinformatic Analyses

Sequences from all transcripts were obtained using a Perl script from transdecoder (https://github.com/TransDecoder/TransDecoder/releases), which uses the coordinates from the merged GTF transcript file of all treatments to extract the sequences from the reference genome (https://plants.ensembl.org/Brassica_napus/Info/Index). Annotation of transcripts was performed with BLASTx against the *A. thaliana* protein database release 10 (TAIR10) using a maximum of 20 hits and an e-*value* of 1e^−10^. The XML BLAST output was loaded to BLAST2GO (Conesa et al., 2005; Conesa and Stefan, 2008) to obtain a consensus annotation for each transcript derived from the 20 top hits. The top *A. thaliana* ID (AtID) was also stored in the database for further analyses.

To find general patterns of gene expression response, we performed functional categorization of genes that were significantly differentially expressed with log_2_-fold changes >1 (upregulated) and ≤1 (downregulated) for each time-point and genotype. The respective AtIDs were used as input for functional enrichment analysis using DAVID (Database for Annotation, Visualization and Integrated Discovery) (Huang et al., 2007; Huang et al., 2009). DAVID was set to obtain gene ontology functional enrichment analysis (biological process level 3), protein domain enrichment analysis, and KEGG pathway enrichment analysis. All transcript AtIDs from the RNA-seq study were used as the background population and a minimum count of five hits was established to find enriched categories bearing an EASE score <0.05. Enriched categories used to create heatmaps were selected after a Benjamini correction for multiple comparisons (*p-*value <0.05). Heatmaps were built with the matrix visualization software Morpheus (https://software.broadinstitute.org/morpheus/) using the corrected *p*-values for enriched gene ontology (GO) biological process ‘level 3' categories, Interpro protein enriched domains, and KEGG enriched pathways.

Log_2_-fold expression values of genes from selected enriched categories from DAVID were used to observe changes through the time-course for both genotypes using Multi-Experiment Viewer (MeV4.9) (Saeed et al., 2003). Hierarchical clustering for genes was applied using Pearson correlation and average linkage. Sub-clusters were generated when necessary to display co-regulated genes.

To find genes with similar or divergent regulation between treatments we used the online tool jvenn (Bardou et al., 2014).

## Results and Discussion

### Disease Development

We compared the anatomical changes that occurred in roots of ‘Laurentian’ (resistant) and ‘Brutor’ (susceptible) following inoculation with *P. brassicae*. The differential response of these hosts was first noted when they were inoculated with resistance-breaking field isolates of the clubroot pathogen recovered from canola crops in Alberta, Canada, in 2013 (Strelkov et al., 2016a). These isolates are classified as pathotype 5 on the differentials of Williams (1966) or as pathotype 5X on the CCD Set (Strelkov et al., 2018). This pathotype is now one of several ‘new’ resistance-breaking pathotypes of concern for canola farmers in western Canada (Strelkov et al., 2016a).

**Figure 1:**
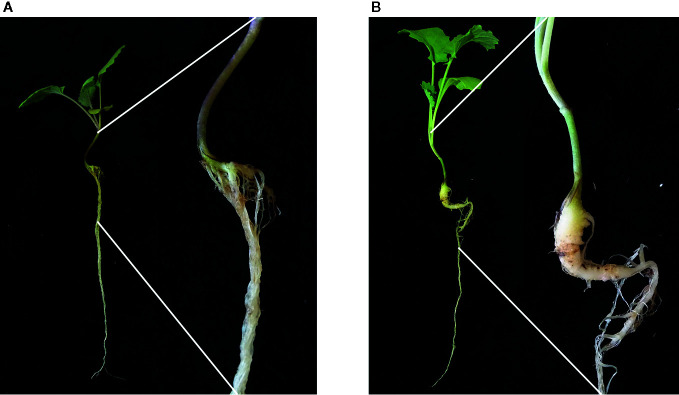
Root gall formation following inoculation with pathotype 5× of *Plasmodiophora brassicae*. No or only incipient galls were observed on the moderately resistant host ‘Laurentian’ **(A)** 21 days after inoculation (dai), while larger and more developed galls had formed on the susceptible host ‘Brutor’ **(B)** at the same time-point.

On the susceptible ‘Brutor’, root galls were visible as early as 14 dai, while no galls were observed on the resistant host ‘Laurentian’ at this time-point. At 21 dai, all ‘Brutor’ plants had developed large galls, while small galls were observed on half of the ‘Laurentian’ seedlings (**Figure 1**). These results indicate that clubroot develops more rapidly and severely on the susceptible genotype. Nonetheless, while there was a differential response between the host genotypes to pathotype 5X, clubroot severity on ‘Laurentian’ was greater than previously reported (Strelkov et al., 2018). Hence, while ‘Laurentian’ showed some resistance to pathotype 5X, it was not immune.

**Figure 2:**
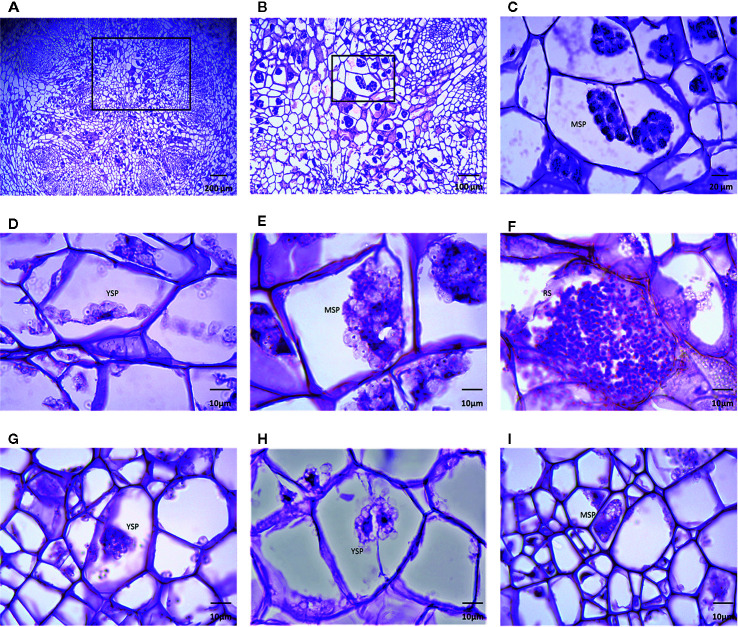
*Plasmodiophora brassicae* infection on roots 21 dai. A cross-section through a large root gall of *Brassica napus* ‘Brutor’ **(A)**, showing the presence of secondary plasmodia, which are visible in more detail under higher magnification **(B, C)**. Young and advanced secondary plasmodia were also visible in other sections of the susceptible ‘Brutor’ **(D–F)**, as well as in the resistant *B. napus* ‘Laurentian’ **(G–I)**. Mature secondary plasmodia (MSP), young secondary plasmodia (YSP), resting spores (RS).

Roots of both hosts were stained with Haematoxylin-Eosin. At 14 dai, ‘Laurentian’ showed few or no signs of infection in the examined cells, while ‘Brutor’ presented what appeared to be young secondary plasmodia. However, at 21 dai, different stages of plasmodial development could be observed inside the cortical cells of both genotypes (**Figure 2**).

### RNA-Seq Data Analysis and Validation

RNA-seq was performed to detect contrasting gene expression patterns between the resistant and susceptible genotypes over a time-course that covered secondary infection by *P. brassicae*. Sequencing of three biological replicates per each inoculated or control treatment for each of ‘Laurentian’ and ‘Brutor’ over three time-points (7, 14 and 21 dai) resulted in over 30 million reads on average per treatment. While ANOVA showed no significant differences in the amount of mapped reads among all treatments (*p =* 0.645), there was a trend showing that the percentage of mapped reads was always lower for the inoculated treatments than for the control treatments (**Supplementary Table S2**). Also, the percentage of mapped reads for the inoculated condition was always lower for ‘Brutor’ than for ‘Laurentian’, and decreased drastically at 21 dai for both hosts, although to a larger extent for ‘Brutor'. These trends are consistent with an increasing number of *P. brassicae* cells colonizing the root tissues of both genotypes (part of the sequenced reads come from *P. brassicae* and therefore are not mapped to *B. napus*).

**Figure 3:**
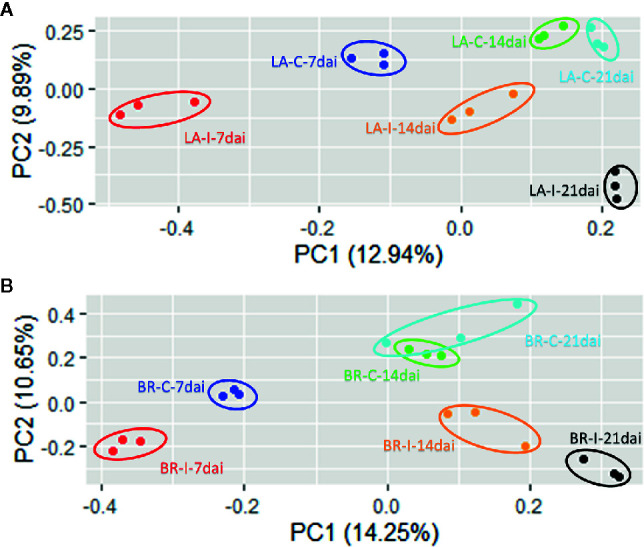
Principal Component Analysis (PCA) plots for RNA-seq data in each genotype. The three biological replicates corresponding to each treatment (control vs. inoculation) in the three time points tested (7, 14 and 21 dai) show consistent clustering for both host genotypes, *Brassica napus* ‘Laurentian’ and ‘Brutor’. **(A)** Laurentian PCA plot. **(B)** Brutor PCA plot. ID codes: LA, Laurentian; BR, Brutor; I, Inoculated; C, Control; number after I or C stands for number of days after inoculation (dai).

The reads were mapped to the *B. napus* reference genome and corresponding gene models, to find expression patterns in existing and novel genes. Fragments Per Kilobase per Million mapped reads (FPKM) values of 105,538 predicted transcripts were used for PCA to test the consistency of the biological replicates. Principal components analysis in each genotype showed the expected clustering of the three replicates from each treatment in the two host genotypes; only one control replicate of ‘Brutor’ at 21 days seemed closer to the replicates from the 14 dai control samples from the same genotype. A larger biological variability among samples can be due to uncontrolled environmental conditions or inherent variations of development among sampled plants. Nevertheless, there was always clear separation between inoculated and control samples in each host and time-point, demonstrating that the pattern of expression is influenced by the treatment and comparisons we established (**Figures 3A, B**). A second PCA analysis was performed to compare control samples from the two genotypes, separated from inoculated samples from the two genotypes. The analysis revealed that control samples were well separated in the first component between genotypes and that the samples from the same day in the two genotypes corresponded in the second component (**Supplementary Figure S1**). The inoculated samples were also separated similarly to the control samples in the first component, but there was separation between the samples of the two genotypes for the corresponding day in the second component. This could indicate some matching regulation between the two genotypes in different time-points, as discussed below.

Of 105,538 predicted transcripts, 85,956 (81.44%) were annotated using the TAIR 10 database. After correcting for multiple comparisons, 5,538, 2,747 and 4,349 genes were regulated at 7, 14 and 21 dai, respectively, for ‘Laurentian’, and 3,834, 3,415 and 6,570 for ‘Brutor’. The number of regulated genes for ‘Laurentian’ was higher than for ‘Brutor' at 7 dai, and there were more upregulated than downregulated genes for both hosts at this time-point. At 14 and 21 dai, the number of downregulated genes was greater than the number of upregulated genes in both hosts; ‘Brutor’ showed the largest number of regulated genes at 21 dai, with most being downregulated (**Table 1**). In the clubroot susceptible *A. thaliana* ecotype ‘Columbia' (Col), an increased number of genes in shoots and roots were regulated at later time-points of infection (Irani et al., 2018), and in *B. rapa* ssp*. pekinensis*, a larger number of genes was regulated in the susceptible interaction when compared with the resistant interaction at 30 dai (Jia et al., 2017). To increase the stringency of downstream analyses, we generated an arbitrary cut-off for significant differentially expressed genes at log_2_-fold changes >1 and ≤1 for up and downregulated genes, respectively (**Table 1**).

**Table 1:** Significant differentially expressed genes in both genotypes throughout the time course, without cut-off (up or down) or with log_2_-fold change >1 or ≤1.

Genotype	Harvest	Up	Down	Up (log_2_-fold ≥1)	Down (log_2_-fold ≤1)
**‘Laurentian'**	***7 dai***	2,946	2,592	1,733	1,541
	***14 dai***	1,237	1,510	741	802
	***21 dai***	1,570	2,779	977	1,907
**‘Brutor'**	***7 dai***	1,936	1,898	1,087	1,055
	***14 dai***	1,696	1,719	786	975
	***21 dai***	1,221	5,349	747	3,596

dai, days after inoculation.

To validate the trends in DEGs detected *via* the RNA-seq analysis, we evaluated 10 target genes across both genotypes by qRT-PCR analysis. The genes selected were differentially expressed and significant in at least one host, in at least one time-point. Comparison of log_2_-fold changes between the RNA-seq and qRT-PCR data showed correlations of >0.8 for the three time-points in the two genotypes (**Supplementary Table S3**).

### Major Transcriptional Changes in the Resistant and Susceptible Interactions

**Figure 4:**
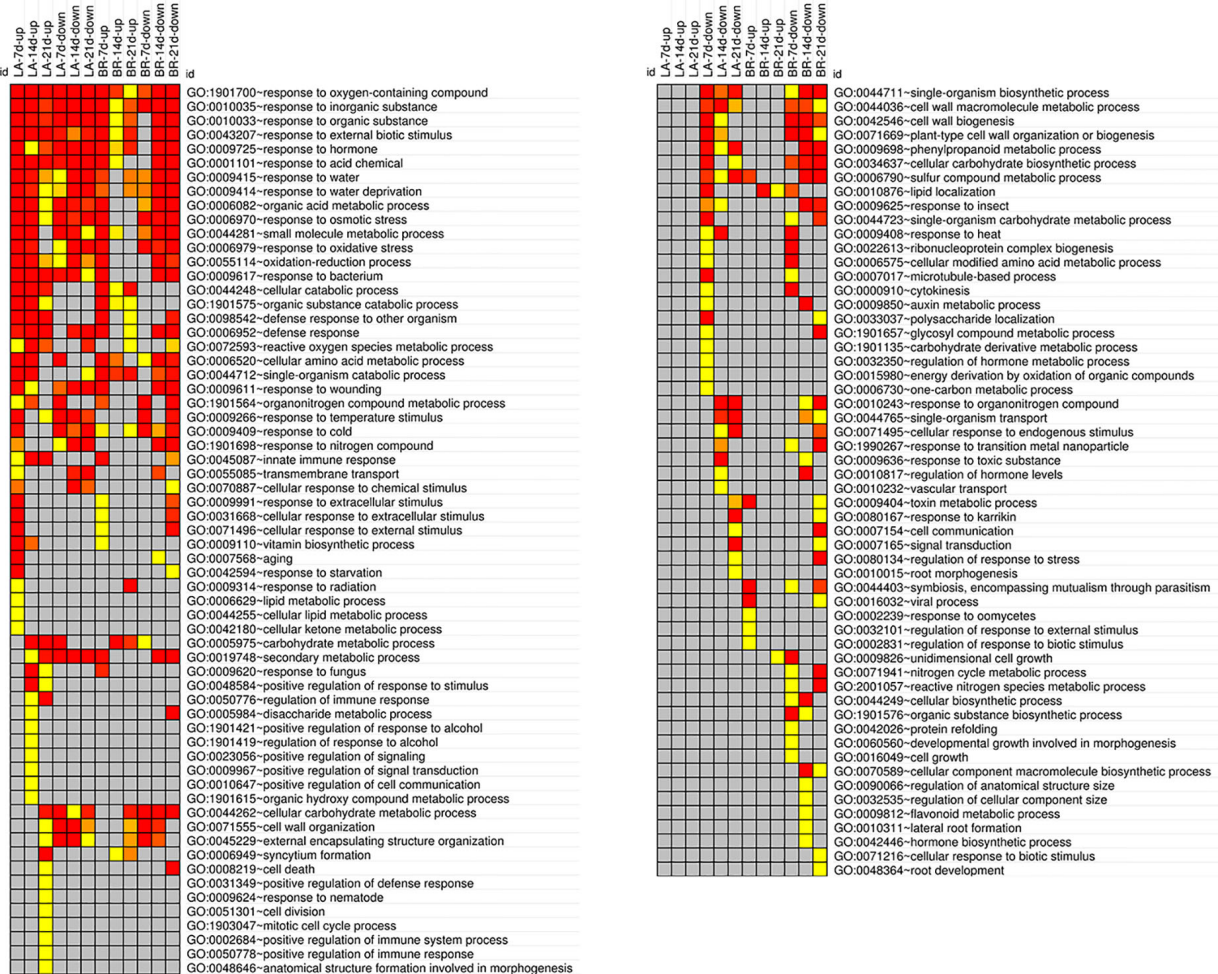
DAVID GO functional category enrichment analysis. Gene ontology (GO) enriched biological process ‘level 3' functional categories are shown across three time-points (7, 14 and 21 days) for upregulated (up) and downregulated (down) genes in the resistant and susceptible *Brassica napus* genotypes, ‘Laurentian' (LA) and ‘Brutor' (BR), respectively. Significantly enriched categories are color-coded (red more significant) according to the EASE score, which constitutes a modified Fisher's exact test (Huang et al., 2009). Most significant EASE value = 2.94E−27, least significant EASE value = 4.95E−02. Non-enriched bins are color coded gray.

Functional enrichment analysis with DAVID using significant differentially expressed genes (log_2_-fold changes >1 or ≤1) across all treatments identified 119 ‘level 3’ GO biological process enriched functional categories (**Figure 4**), 73 enriched Interpro domains and 28 enriched KEGG pathways (**Supplementary Figure S2**).

### General Stress Response

A wide scale stress response is expected upon pathogen challenge. Functional categories where all or most bins were regulated through the time-course for the two host genotypes included response to oxygen-containing compounds, response to inorganic/organic substances, response to external biotic stimulus, response to hormones, response to acid chemicals, response to osmotic stress and water deprivation, response to oxidative stress/oxidation-reduction, and response to bacterium (**Figure 4**). The enrichment of the response to hormone category supports the central role that hormones play in this pathosystem, where growth hormones like auxins and cytokinins have been directly involved in the hypertrophy and hyperplasia of host tissues invaded by *P. brassicae* (Ludwig-Müller, 2009; Ludwig-Müller et al., 2009). Drought stress enrichment likely reflects the inhibition of water uptake and transport during secondary infection, due to disruption of the roots and vascular system (Ludwig-Müller, 2009). Finally, the oxidative response is part of a defense mechanism that includes the activation of oxidative burst-related enzymes that act as signalling molecules, but can also have direct antimicrobial effects or result in localized cell death. This behavior is supported by several studies of the *P. brassicae*–Brassicaceae interaction (Agarwal et al., 2011; Jubault et al., 2013; Zhang et al., 2016b; Zhao et al., 2017).

### Immunity in Response to *P. brassicae* Is Regulated Extensively by Salicylic Acid

**Figure 5:**
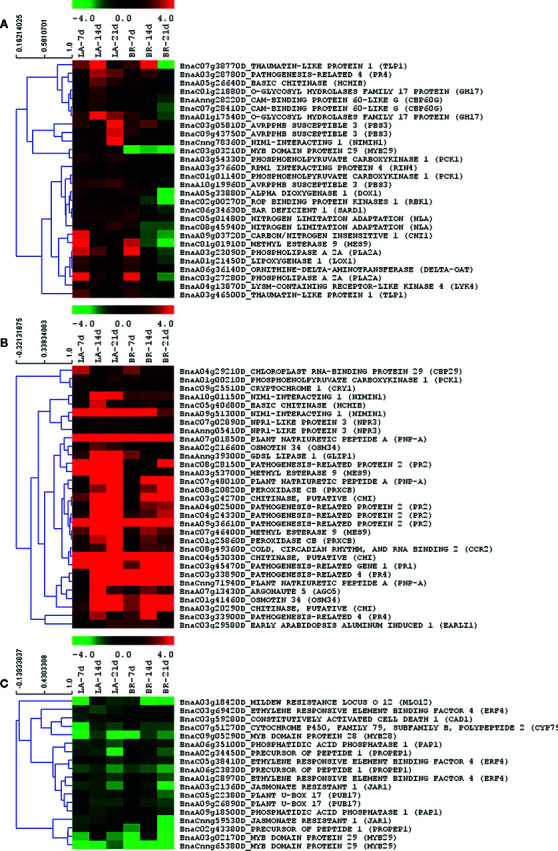
Immunity-related gene regulation. Genes from this functional category clustered according to expression patterns. Heatmaps were built with MeV with log_2_-fold expression limits of 4 (red) to −4 (green). Hierarchical clustering of gene expression patterns was performed using Pearson correlation. Clusters **(A–C)** represent different trends in gene expression co-regulation.

Innate immune responses in plants can be activated in roots by pathogen-associated molecular patterns (PAMPs) leading to PAMP-triggered immunity (PTI), or by effectors leading to effector-triggered immunity (ETI) (Miller et al., 2017). Molecule recognition usually leads to an oxidative burst (via the production of reactive oxygen species (ROS)), where calcium takes part in a signal transduction cascade to modulate transcription factors that in turn activate defense responses (Stael et al., 2015). In our study, a clear distinction was observed in the innate immune response functional category. This category was enriched for upregulated genes in ‘Laurentian’ at 7, 14 and 21 dai (**Figure 4**). In contrast, ‘Brutor’ showed enrichment for this category only for upregulated genes at 7 dai and for downregulated genes at 21 dai. While the expression level of many genes in this functional category followed the pattern of enrichment (**Figure 5A**), other immune response genes where mostly upregulated (**Figure 5B**) or downregulated (**Figure 5C**) for both genotypes throughout the time course.

Many of the genes that displayed increased transcriptional levels in ‘Laurentian’ at 7, 14 and 21 dai, and in ‘Brutor’ at 7 dai were key receptors/signalling genes or were related to salicylic acid (SA)-mediated responses (**Figure 5A**). Other immunity-related genes displayed more heterogeneous patterns of regulation (**Supplementary Figure S3**).

Kinase regulators are key in signal transduction of pathogen signals. Among these, *RBK1* (ROP-binding kinase 1) showed sustained expression in the resistant host ‘Laurentian’ (**Figure 5A**). RBK1 is phosphorylated by the mitogen-activated protein kinase 1 (MPK1), which negatively influences auxin-dependent cell expansion (Enders et al., 2017). Therefore, RBK1 may be a key component in downstream regulation of auxin-dependent responses.

Downstream of signal transduction, the functionally redundant transcription factors involved in SA signalling, Calmodulin-Binding Protein 60g (*CBP60g*) and Systemic-Acquired Resistance Deficient 1 (*SARD1*) (Wang et al., 2011a), were also regulated in the current study (**Figure 5A**). Two transcripts corresponding to *CBP60g* had their highest upregulation at 14 dai in ‘Laurentian’, while a transcript matching *SARD1* was upregulated throughout the time course (**Figure 5A**). For the susceptible genotype both genes were upregulated at 7 dai and downregulated at 14 and 21 dai. The susceptibility of *A. thaliana* to *Pseudomonas syringae* was enhanced when these two genes were mutated, with a concomitant reduction of both, salicylic acid (SA), and SA-mediated marker genes like *PR1* (Wang et al., 2011a). Two genes involved in regulation and transport of SA, isochorismate synthase (*ICS*) and enhanced disease susceptibility 5 (*EDS5*), act downstream of *CPB60g/SARD1* (Zhang et al., 2010; Wang et al., 2011a). Isochorismate synthase is translocated to the chloroplasts and is involved in the transformation of chorismite into isochorismate, a precursor of SA, while *EDS5* allows the translocation of the SA produced in the chloroplast into the cytoplasm. One transcript corresponding to gene *ICS2* (BnaC08g18420D), which is redundant in function with *ICS1*, was highly expressed across the three time-points in ‘Laurentian’ (**Supplementary Table S4**), while expression of *EDS5* peaked at 21 dai in this genotype (**Supplementary Figure S3B**). Once SA accumulates in the cytoplasm, it is regulated *via* different mechanisms. For example, the pool of SA can be increased when cytoplasmic methyl esterases transform MeSA into SA (Dempsey et al., 2011). Transcripts matching methyl esterase 9 (*MES9*) showed high expression in both genotypes, with a stronger regulation in ‘Laurentian’ (**Figures 5A, B**). *MES9* from *B. oleracea* was also upregulated in both resistant and susceptible genotypes upon *P. brassicae* infection, but with a stronger regulation in the susceptible genotype (Manoharan et al., 2016). Another gene, *PBS3* (AvrPphH susceptible 3), promotes accumulation of SA by suppressing proteins that conjugate SA to amino acids (Lee et al., 2007; Nobuta et al., 2007; Dempsey et al., 2011). Three transcripts of *PBS3* were predominately upregulated throughout the time course in ‘Laurentian’ (**Figure 5A**). This gene confers resistance to the biotrophic pathogen *P. syringae* and acts synergistically with *NPR1* upstream of SA to mediate defense responses (Lee et al., 2007; Nobuta et al., 2007).

Regulation of responses to different levels of SA are mediated by an array of different genes. For example the *EDS1* (enhanced disease susceptibility 1)*-PAD4* (phytoalexin deficient 4)*/SAG101* (senescence-associated gene 101) complex (Wagner et al., 2013) is involved in a positive feedback loop for SA accumulation that includes *ICS* expression (Seyfferth and Tsuda, 2014). While our results indicated regulation of transcripts corresponding to these genes, their transcriptional levels did not produce a clear trend for either host genotype or time-point (**Supplementary Figure S3C** and **Supplementary Table S4**). Another family of genes, the non-expressor of PR (*NPR)* genes, are involved in regulating SA levels. NPR1 is the main transducer of SA signals, interacting as a co-factor of transcription factors to alter defense (Cao et al., 1997; Dong, 2004). NPR1interacts specifically with TGA factors to bind the promoter of the important defense marker *PR1* (Chen et al., 2016b), to modulate its activation (Weigel et al., 2005). While our study did not find regulation of the transcript corresponding to *NPR1, PR1* was highly upregulated throughout the time course in both host genotypes (**Figure 5B**). A second transcript of the *NPR* family, *NPR3*, was significantly upregulated at 14 and 21 dai in ‘Laurentian’ and at 7 dai in ‘Brutor’ (**Figure 5B**). NPR3 and its paralog NPR4 serve as adaptors for a CUL3 ligase, targeting NPR1 for degradation depending on SA concentration; with low concentrations promoting an NPR1-NPR4 association and with high SA concentrations promoting an NPR1-NPR3 association (Fu et al., 2012; Moreau et al., 2012). Another mechanism to regulate SA through degradation is provided by the nitrogen limitation adaptation (*NLA*) gene. This gene acts as a negative regulator of SA accumulation upon *P. syringae* infection, and seems to be independent of the *ICS1*-mediated SA accumulation (Yaeno and Iba, 2008). It was argued that *NLA* codes for a ring-type ubiquitin E3 ligase, and mutations in this gene result in excessive levels of SA (Yaeno and Iba, 2008), suggesting that NLA is involved in SA-mediated immune response regulation *via* ubiquitination. Transcripts of *NLA* were downregulated in ‘Brutor’ at 14 and 21 dai and upregulated in ‘Laurentian’ throughout the time-course (**Figure 5A**). Keeping SA levels consistent (not under- or over-regulated) may be necessary for the basal defense response of the resistant ‘Laurentian’, hence the observed upregulation of *NLA* throughout the time course by this host. Supporting this view, silencing of *NLA via* microRNA activation increased susceptibility of *A. thaliana* to a cyst nematode (Hewezi et al., 2016). One more gene that could have a similar function to *NLA* was the carbon/nitrogen insensitive 1 gene (*CNI1*). This gene was upregulated in ‘Laurentian’ throughout the time course and downregulated in ‘Brutor’ at 14 and 21 dai (**Figure 5A**). *CNI1* is also known as Arabidopsis toxicos en levadura 31 (*ATL31*), a RING-type ubiquitin ligase that is upregulated by bacterial elicitors and increases plant resistance when overexpressed (Maekawa et al., 2012). We speculate that the resulting SA-immune response inferred from our differential expression analysis (especially in the resistant genotype) is potentially accompanied by increasing SA levels, which can cause the activation of genes that fine tune the SA-mediated plant defense.

**Figure 6:**
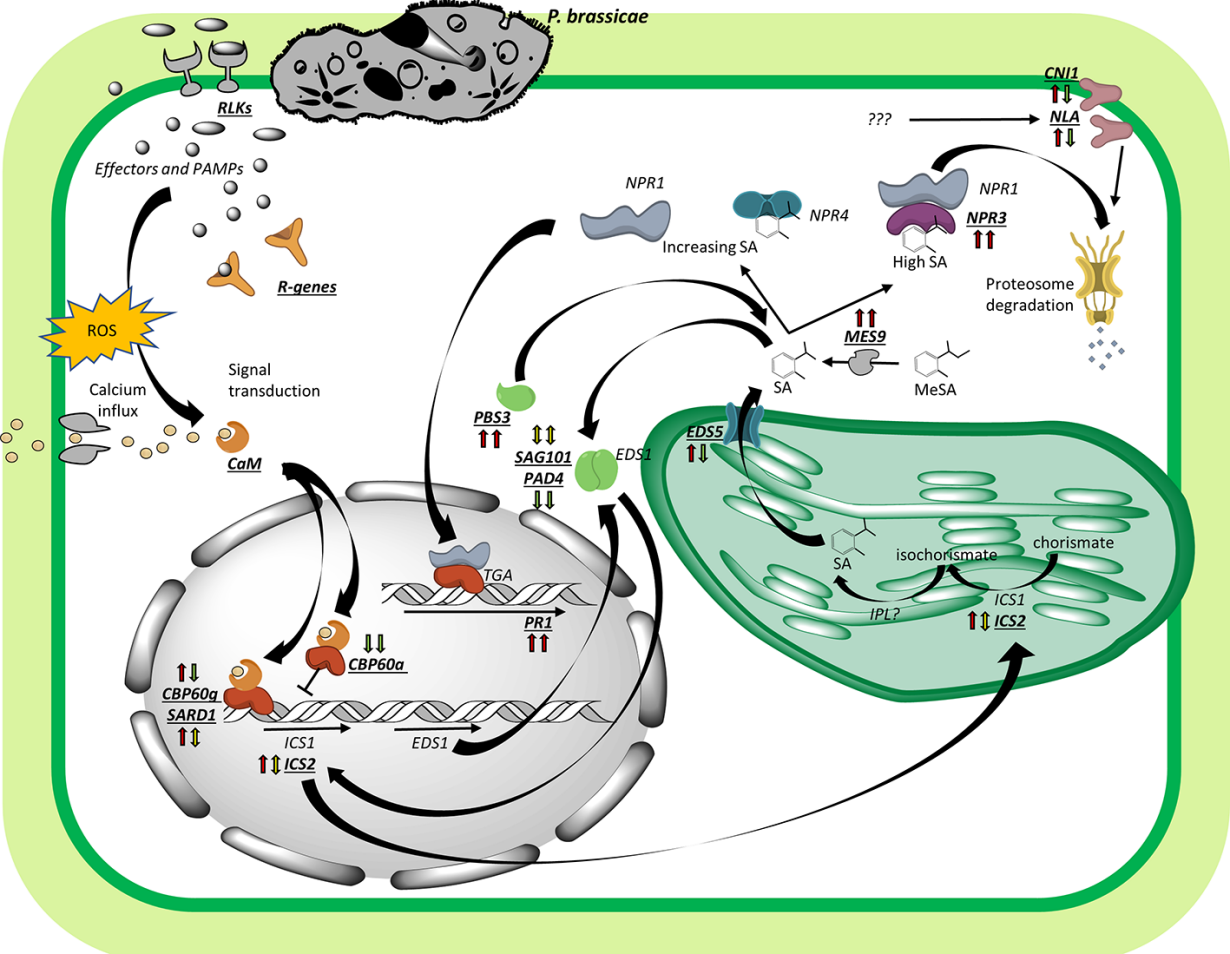
Model of SA-mediated immunity. SA-related genes (bold-italics-underlined) were regulated differentially in the interaction between *Plasmodiophora brassicae* and the *Brassica napus* hosts ‘Laurentian' and ‘Brutor', as indicated by the left and right arrows, respectively, next to each gene (red: upregulated, green: downregulated, yellow: mixed/undetermined regulation; expression trend of the arrows is based on all time-points and associated transcripts of the specific gene with log2-fold changes >1 or ≤1). Detection of PAMPs and effectors by RLKs and R-genes triggers a change in calcium status and an oxidative burst leading to signal transduction. Calcium sensor proteins (CaM) can bind transcription factors such as CBP60g/SARD1 or CBP60a, which positively or negatively regulate expression of genes like ICS. ICS is translocated to the chloroplast to transform chorismate into isochorismate, a SA precursor. SA is then translocated into the cytoplasm by the EDS5 transporter, where additional SA can be synthesized from methyl salicylate thanks to the action of MES9. Under low SA levels, NPR1 binds NPR4, but as SA increases NPR4 binds SA and dissociates from NPR1, allowing NPR1 to fine-tune the response of defense genes (like PR1) *via* its interaction with transcription factors (TGA). If SA levels keep increasing, NPR3 binds SA and associates with NPR1, promoting NPR1 degradation *via* the proteasome. At the same time, feedback loops between associated homomeric and heteromeric complexes between PAD4/SAG101 and EDS1, and the gene PBS3, regulate SA levels. Finally, NLA/CNI1 help to control the levels of SA tagging unknown genes for degradation *via* the proteasome. Abbreviations: PAMPs (pathogen-associated molecular patterns), ROS (reactive oxygen species), RLKs (receptor like kinases), R-genes (resistance genes), CaM (calcium-modulated protein/calmodulin), CBP60g (calmodulin-binding protein 60g), SARD1 (systemic-acquired resistance deficient 1), CBP60a (calmodulin-binding protein 60a), ICS1 (isochorimate synthase 1), ICS2 (isochorismate synthase 2), IPL (isochorismate pyruvate lyase), EDS5 (enhanced disease susceptibility 5), MES9 (methyl esterase 9), NPR1 (non-expressor of pathogenesis related genes 1), NPR3 (non-expressor of pathogenesis related genes 3), NPR4 (non-expressor of pathogenesis related genes 4), NLA (nitrogen limitation adaptation), CNI1 (carbon/nitrogen insensitive 1), TGA (TGA-binding bZIP transcription factors), PR1 (pathogenesis-related protein 1), EDS1 (enhanced disease susceptibility 1), SAG101 (senescence-associated gene 101), PAD4 (phytoalexin deficient 4), PBS3 (AvrPphB susceptible 3).

Evidence of the regulation of immunity through SA in our study is consistent with a typical biotrophic interaction, with longer lasting effects in the resistant but not in the susceptible host. In *A. thaliana*, the partially resistant ecotype Bur-0 activated SA signalling through secondary pathogen infection, while the susceptible Col-0 did not show SA-mediated responses (Lemarie et al., 2015). The exogenous application of SA reduced clubroot symptom development on *B. oleracea* and *A. thaliana* (Lovelock et al., 2013; Lemarie et al., 2015), highlighting the importance of this hormone for defense against *P. brassicae.* A model of SA-mediated immunity responses is presented in **Figure 6**. While many of the genes found in our study are regulated in the same manner in both hosts, key genes are mainly upregulated in the resistant genotype, which supports a stronger SA-mediated immune response in ‘Laurentian’.

### SA and SAR Are Antagonistic to JA/ET in Both Genotypes

After establishing the mechanisms of SA-mediated immunity, we analyzed the role of other DEGs that were also part of the immune response functional category. Transcription of some of these genes is influenced by SA regulation, while others regulate defenses through crosstalk with other hormones.

We found a strong induction of transcripts for plant natriuretic peptides (*PNP*s) in both hosts throughout the time course (**Figure 5B**). PNPs are signalling molecules that respond to biotic and abiotic stresses (Wang et al., 2011b). In response to a *P. syringae* effector, transcriptional regulation of *PNP-A* was shown to be dependent on *EDS1* and therefore related to SAR (Breitenbach et al., 2014). Furthermore, co-expression analysis showed that *A. thaliana PNP-A* expresses together with genes that are involved in SAR, and also induced by SA or analogue molecules (Meier et al., 2008). Among these genes, the transcripts corresponding to *PR1 PR2, NIMIN1* and *WRKY70* were mostly upregulated throughout the time course in both of our hosts (**Figures 5A, B**, **Supplementary Figure S3B**). These genes are important components of plant defense responses and *WRKY70* was shown to specifically control *AtPNP-A* (Meier et al., 2008). The transcription factor *WRKY70* was also shown to be the key regulator of antagonistic responses between SA and JA (Li et al., 2006). Modulated by NPR1, *WRKY70* potentially binds promoters of negative regulators of JA signalling or induces factors that block positive regulators (Li et al., 2006). *WRKY70* was activated in the *A. thaliana* paritally resistant genotype Bur-0 upon *P. brassicae* infection, in parallel with strong activation of SA-mediated defenses and repression of JA-mediated responses (Jubault et al., 2013). The upregulation of *WRKY70* in our study, plus the downregulation of genes like jasmonate resistant 1 (*JAR1*) (**Figure 5C** and **Supplementary Figure S3C**), a gene induced by jasmonate and catalyzer in the generation of active JA (Wasternack and Hause, 2013), support a SA-mediated response which is antagonistic to JA responses.

It is also known that JA acts synergistically with ethylene (ET), usually in response to necrotrophic pathogens (Wasternack and Hause, 2013). In this case, we would also expect an inhibition of ethylene mediated responses in our study. The key marker of JA/ET-mediated defense responses, *PDF1.2* (Plant defensin) (Penninckx et al., 1996), was not regulated in either of the genotypes in our study, while a peptide elicitor of *PDF1.2* (precursor of peptide 1) that is activated by methyl jasmonate and ethylene (Huffaker et al., 2006) was downregulated (**Figure 5C**). Additionally, the ethylene response factor 4 (*ERF4*), which is regulated *via* JA (McGrath et al., 2005; Yang et al., 2005), and *ERF2*, which acts as a transcriptional activator of ethylene responsive genes (Fujimoto et al., 2000), were mainly downregulated (**Figure 5C** and **Supplementary Figures S3A, C**).

### Pathogenesis-Related (PR) Proteins and Susceptibility Factors Contribute to Defense Against Clubroot

PR proteins are generally activated due to induction of SA and SAR. Among these, chitinases and thaumatins have been shown to have a direct effect on pathogen cell walls and membranes, arresting pathogen spread, or producing oligomers that act as signals to initiate reprogramming of defenses (Grover et al., 2012). Most chitinases were mainly upregulated in both genotypes throughout the time course (**Figures 5A, B**), and chitin-binding domains were also enriched for upregulated ‘Laurentian’ genes 21 dai (**Supplementary Figure S2**). Upregulation of these genes was detected in CR *B. rapa*, *B. oleracea* and *B. macrocarpa* (Chen et al., 2016a; Zhang et al., 2016b; Wang et al., 2019). The thaumatin-like protein 1 (*TLP1*) gene, which was upregulated at 7 and 14 dai in both genotypes and downregulated at 21 dai in ‘Brutor’ (**Figure 5A**), has been shown to be involved in defense against nematodes (Hamamouch et al., 2012), organisms which usually cause symptoms in their host resembling clubroot symptoms (e.g. enlarged cells).

Additional PR genes (*PR1, PR2, PR4*) were consistently upregulated in both host genotypes (**Figure 5B**). *PR1, PR2* and *PR5* are known to be SA-responsive, and *PR2* and *PR5* have been shown to be induced during secondary infection by *P. brassicae* of partially resistant *A. thaliana* (Lemarie et al., 2015).

While upregulation of defense genes is commonly seen as a strategy to deter a pathogen, downregulation of certain genes (susceptibility factors) may also contribute to immunity. In *A. thaliana* triple mutants, *mlo2, mlo6* and *mlo12* confer resistance to powdery mildew *via* the activation of multiple basal defense mechanisms (Kuhn et al., 2017). In our study, the mildew resistance locus 12 gene (*MLO12*) had one associated transcript with complete downregulation in all treatments (**Figure 5C**), and a second transcript slightly upregulated at 7 dai in both genotypes (**Supplementary Figure S3A**), while a transcript matching *MLO2* showed a trend similar to the second *MLO12* transcript. Another gene, *CAD1* (constitutively activated cell death 1) negatively regulates SA-mediated responses, with knockouts of the gene resulting in activation of *PR1* (Asada et al., 2011). Downregulation of this gene in our study, mainly in the case of ‘Laurentian' (**Figure 5C** and **Supplementary Figure S3C**), supports steady SA-mediated immunity.

### Similar Symptoms, Similar Responses: *P. brassicae* and Nematodes Activate Comparable Pathways

**Figure 7:**
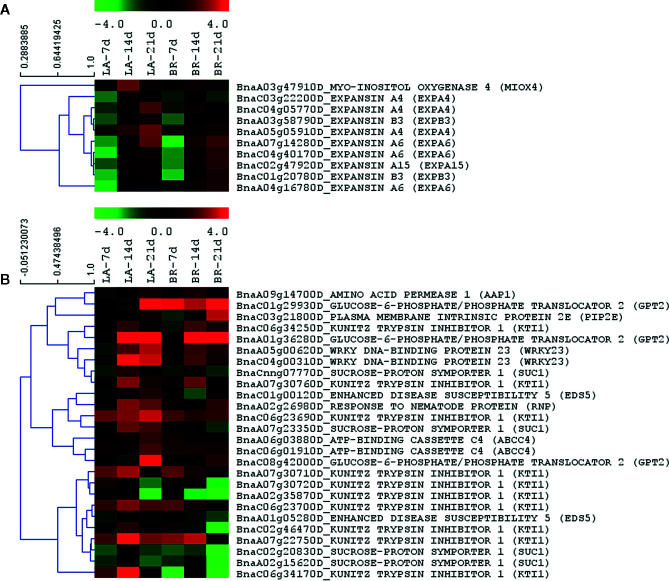
Nematode-like gene regulation responses. Two functional categories in the interaction between *Plasmodiophora brassicae* and *Brassica napus* indicated responses similar to nematode infections: **(A)** Syncytium formation. **(B)** Response to nematode. Heatmaps were built with MeV with log_2_-fold expression limits of 4 (red) to −4 (green). Hierarchical clustering of gene expression patterns was performed using Pearson correlation.

The formation of a syncytium—an enlarged multinucleated cell mass—is usually triggered by parasitic nematode infection of plants (Rodiuc et al., 2014). The formation of syncytia shares some parallels with the root swelling and enlarged cells that are associated with clubroot development. Cell hypertrophy and cell wall modification constitute common processes for both the clubroot interaction and nematode infections (Rodiuc et al., 2014; Chen et al., 2016a). Root-knot nematodes induce galls with giant cells, where mitosis progresses without cytokinesis to generate the syncytium, whereas cell enlargement and accelerated cell division occur during clubroot development. These processes result in disruption of water and nutrient transport through the plant, generating sinks that favor parasite development. In the present study, the category of syncytium formation was enriched for upregulated genes at 21 dai for ‘Laurentian’ and at 14 and 21 dai for ‘Brutor’ (**Figure 4**). Many of the transcripts within this functional category matched expansins that were upregulated in both hosts, especially at 14 and 21 dai (**Figure 7A**).

‘Laurentian’ showed an enrichment of upregulated genes only at 21 dai for the category response to nematode (**Figure 4**). Some of the *B. napus* transcripts that matched the *A. thaliana* IDs from this functional category suggest a potential effector-related manipulation of the host (Irani et al., 2018). In a susceptible interaction between *A. thaliana* and *P. brassicae*, the highest gene expression corresponded to a glucose-6-phosphate/phosphate translocator (*GPT2*) (Irani et al., 2018). This is the exact same glucose transporter found in our study, which matched three transcripts showing different levels of upregulation in both hosts throughout the time course (**Figure 7B**). Amino acids are also important for pathogen development, and an amino acid permease (*AAP1*) displayed higher expression at 21 dai in ‘Laurentian’ and at 14 dai in ‘Brutor’ (**Figure 7B**). In *A. thaliana* plants infected by the cyst nematode *Heterodera schachtii*, *AAP1* along with other five *AAP* genes were upregulated to provide amino acids in the sink syncytial tissue, where the nematodes were developing (Elashry et al., 2013); mutants of these genes decreased the incidence of infection. Therefore, the expression of *GPT2* and *AAP1* in *P. brassicae* infected plants could provide amino acids and carbohydrates for pathogen nutrition and metabolic activity.

Another interesting gene in the ‘response to nematode' functional category was *WRKY23*. Transcripts from this gene were upregulated at 14 and 21 dai in both host genotypes, and to a lesser extent in ‘Brutor’ at 7 dai (**Figure 7B**). This transcription factor has been linked to polar transportation of auxin (Prat et al., 2018), a hormone that is at the core of increased host cell growth due to *P. brassicae* infection (Ludwig-Müller, 2009; Ludwig-Müller et al., 2009). Knocking down of this gene in *A. thaliana* resulted in reduced levels of infection by a pathogenic nematode (Grunewald et al., 2008). Since auxin is key for the establishment and colonization of both nematodes and *P. brassicae*, we hypothesize that both interactions trigger similar host responses.

### Cell Wall Metabolism: Transcriptional Repression of Specific Cell Wall Genes can Contribute to Plant Defense

Cell wall modification is a common mechanism of defense against pathogen progression, and alterations to the cell wall structure may contribute to stopping disease. Two functional categories related to cell wall organization or biogenesis were enriched for downregulated genes at 7 and 14 dai in ‘Laurentian’ and at 7, 14 and 21 dai in ‘Brutor’ (**Figure 4**). Genes related to cell wall organization or biogenesis were upregulated at 7 dai and downregulated at 14 dai in resistant wild cabbage (Zhang et al., 2016b), and in *A. thaliana* roots challenged with the clubroot pathogen, the cell wall organization functional category was enriched with downregulated genes (Irani et al., 2018).

While most genes related to cell wall metabolism were downregulated in both hosts throughout the time course (**Supplementary Figure S4A**), other genes including expansins and xyloglucan endotransglucosylase/hydrolase (*XTHs*) showed a mixed response (**Supplementary Figures S4B, C**). Expansins and *XTHs* are important genes promoting cell growth through cell wall loosening, which favors *P. brassicae* infection (Devos et al., 2005; Siemens et al., 2006). These genes have been found to be upregulated in susceptible interactions (Agarwal et al., 2011; Irani et al., 2018), while symptomless *B. oleracea* plants and resistant *A. thaliana* interactions show downregulation of these genes (Jubault et al., 2013; Ciaghi et al., 2019). Except for a few transcripts annotated as cellulose synthase-like genes (**Supplementary Figure S4C**), cellulose synthases (CESAs) were largely downregulated in both hosts in the current study (**Supplementary Figure S4A**). Other genes involved in cell wall deposition, including cobra-like proteins (*COBL*) and irregular xylem genes (corresponding to CESAs and glycosyltransferases), and genes involved in cell wall lignification like peroxidase 25 (*PRX25*), were also mostly downregulated (**Supplementary Figure S4A**). Cellulose synthases (CESAs) control cellulose deposition in primary and secondary cell walls. Counterintuitively perhaps, *A. thaliana* CESAs mutants have been shown to be resistant to several pathogens (Ellis et al., 2002; Hernandez-Blanco et al., 2007; Malinovsky et al., 2014). The reaction of these mutants seems to be concomitant with a constitutive plant immune response, potentially through reallocation of resources to defense mechanisms. Overall, the regulation of cell wall genes indicates common mechanisms between the susceptible and moderately resistant host genotypes, with little marked differences between the two.

## Contrasting and Shared Gene Expression Leads to Clues Regarding Susceptibility vs. Resistance

### Contrasting Patterns of Gene Expression Between Cultivars at the Same Time Point

We created Venn diagrams for each time-point to identify genes with contrasting expression patterns. These genes can be key regulators and mark differences between susceptible and resistant interactions. Such genes are good candidates for gene editing-based mutagenesis to either validate gene function or increase host resistance. Genes downregulated in ‘Laurentian’ and upregulated in ‘Brutor’ could be susceptibility factors, while genes upregulated in ‘Laurentian' and downregulated in ‘Brutor’ could be involved in resistance.

**Figure 8:**
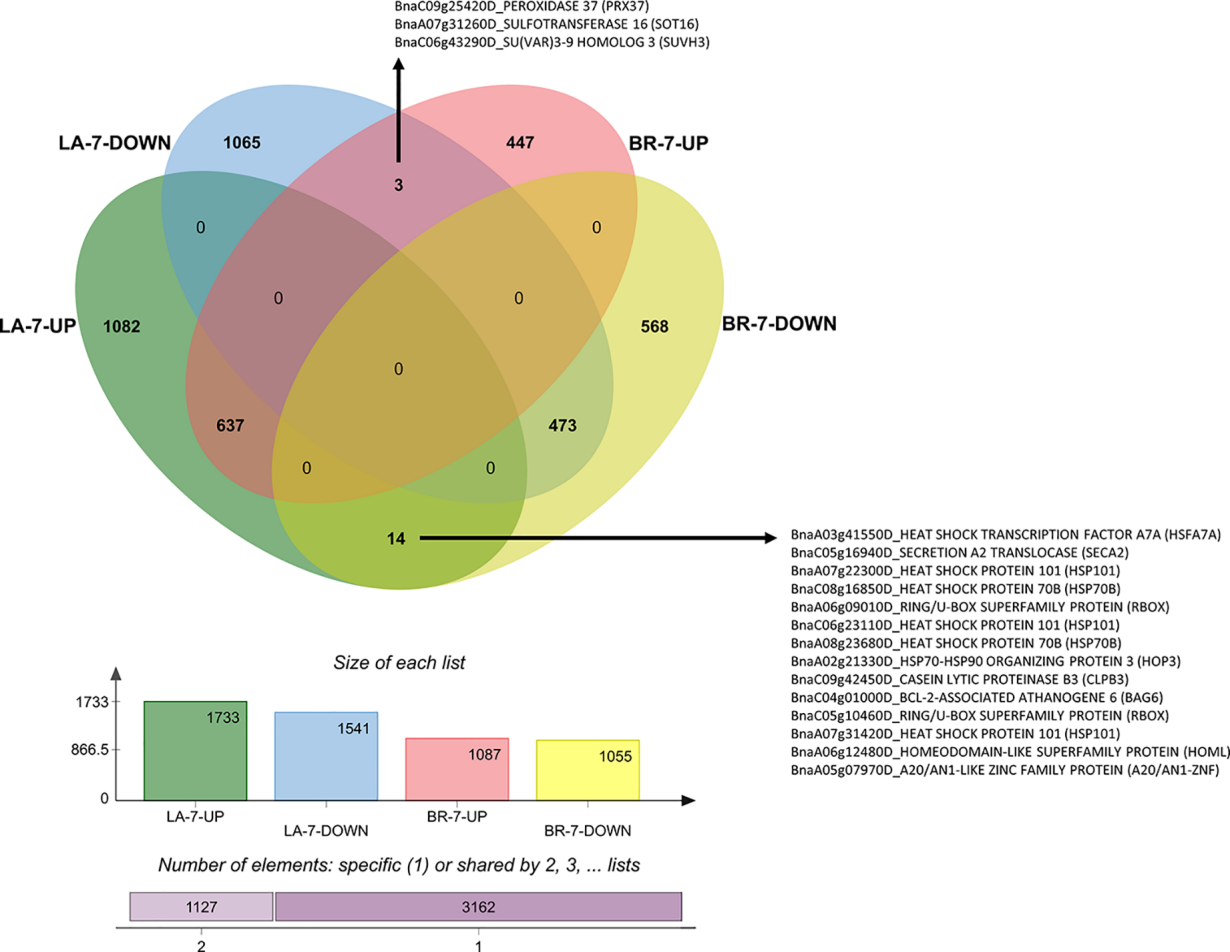
Venn diagram of DEGs 7 dai. The diagram depicts the number of genes with common, opposite, and distinct expression patterns in *Brassica napus* ‘Laurentian' and ‘Brutor' in response to *Plasmodiophora brassicae* pathotype 5X. The annotation of genes that demonstrated opposite expression between the genotypes is shown. LA, Laurentian; BR, Brutor.

At 7 dai, three genes were significantly downregulated in ‘Laurentian’ and upregulated in ‘Brutor’ (**Figure 8**). One of these transcripts corresponded to *PRX37,* a peroxidase potentially involved in cell wall cross-linking *via* ROS generation. This gene was coregulated with *PDF1.2* in an *A. thaliana* overexpressor of the transcription factor DEWAX, which showed increased resistance to *Botrytis cinerea* (Ju et al., 2017).

Most genes upregulated in ‘Laurentian’ and downregulated in ‘Brutor’ at 7 dai matched heat shock proteins (HSPs) (**Figure 8**). HSPs (e.g., HSP90) maintain the tridimensional integrity of proteins including membrane and cytosolic receptors and transductors of pathogenic signals (e.g., RPM1), (Hubert et al., 2003; Park et al., 2015). Complexes including cochaperones (HOP3) and HPS70 and HSP90 may be key to allowing correct protein folding in the ER during periods of high abiotic or biotic stress (Fernández-Bautista et al., 2017). The activation of these genes in the moderately resistant genotype (‘Laurentian’) is notable since, to our knowledge, there are only two reports of regulation of HSPs in this pathosystem (Zhao et al., 2017; Luo et al., 2018).

**Figure 9:**
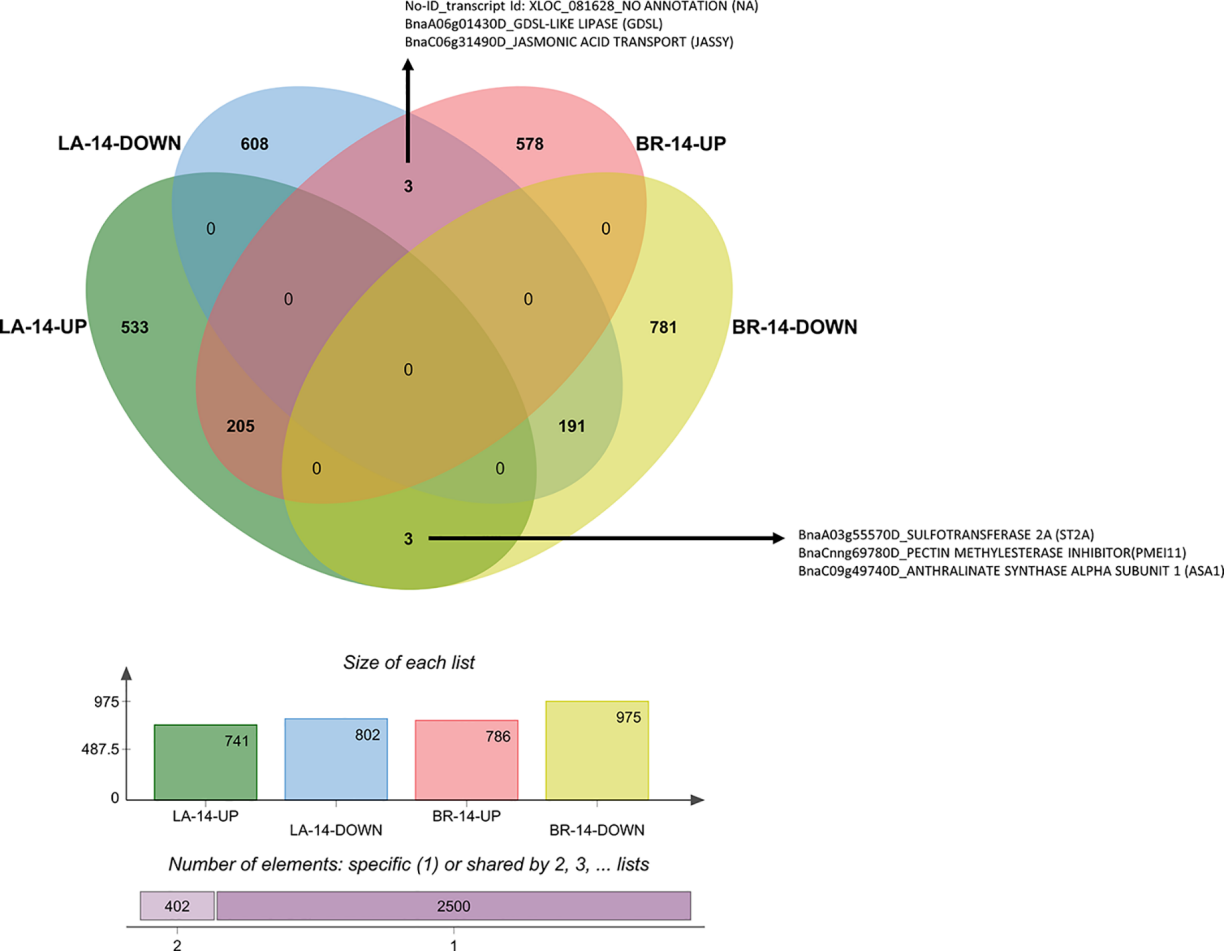
Venn diagram of DEGs 14 dai. The diagram depicts the number of genes with common, opposite, and distinct expression patterns in *Brassica napus* ‘Laurentian’ and ‘Brutor’ in response to *Plasmodiophora brassicae* pathotype 5X. The annotation of genes that demonstrated opposite expression between the genotypes is shown. LA, Laurentian; BR, Brutor.

At 14 dai, the pectin methylesterase inhibitor (*PMEI11*) was upregulated in the resistant host and downregulated in the susceptible one (**Figure 9**). Previously, *PMEI11* was found to be upregulated in response to two necrotrophic pathogens, *A. thaliana* challenged with *B. cinerea* (Lionetti et al., 2017), and *B. napus* infected with *Sclerotinia sclerotiorum* (Zhao et al., 2007). A second gene upregulated in ‘Laurentian’ and downregulated in ‘Brutor’ at 14 dai, anthranilate synthase alpha subunit (*ASA1*), was also regulated in response to *S. sclerotiorum* in the same study (Zhao et al., 2007). Along with *PMEI11*, *ASA1* was one of 12 candidate genes with high expression and linkage to resistance QTLs in the *B. napus–S. sclerotiorum* interaction (Zhao et al., 2007).

**Figure 10:**
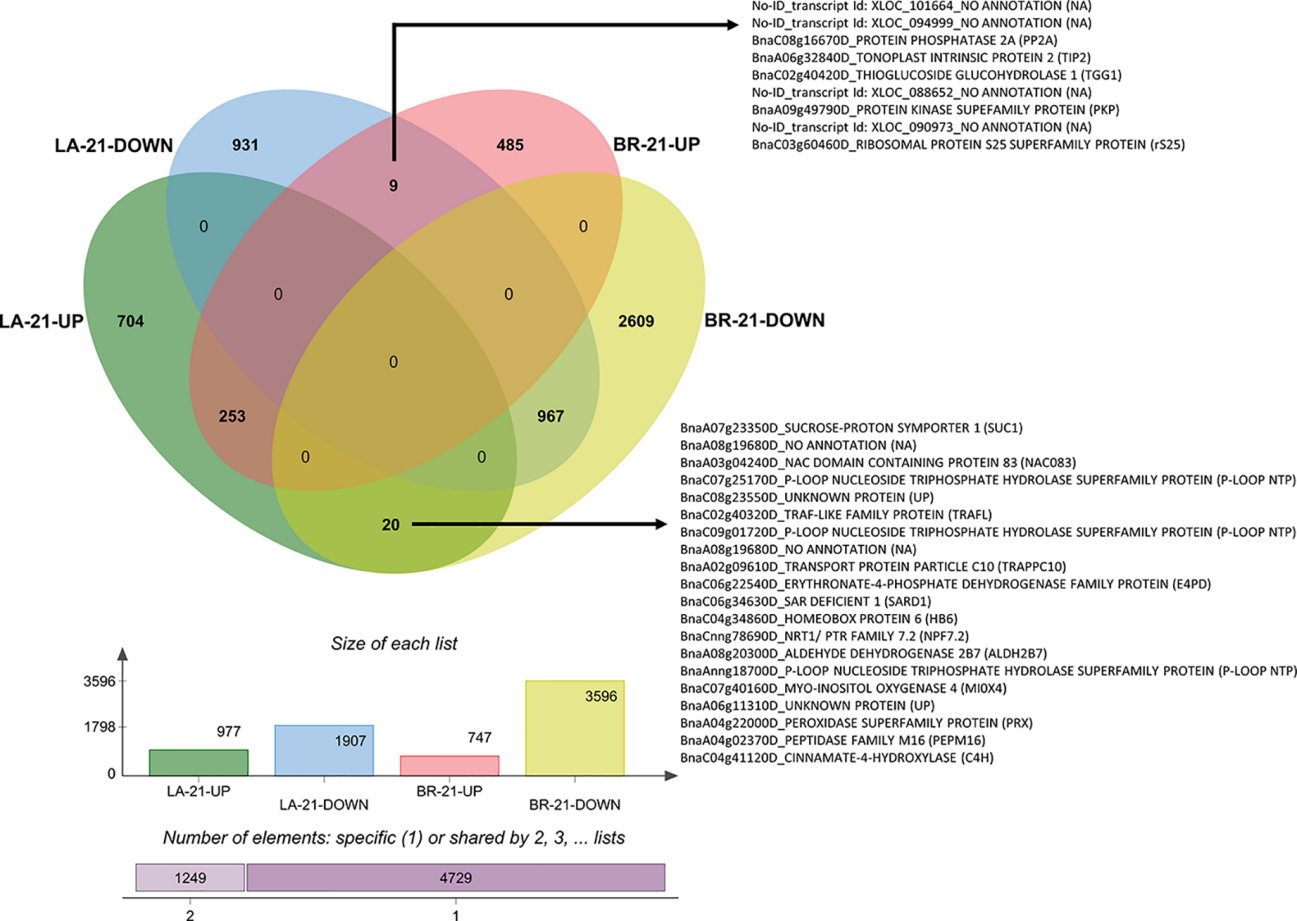
Venn diagram of DEGs 21 dai. The diagram depicts the number of genes with common, opposite, and distinct expression patterns in *Brassica napus* ‘Laurentian’ and ‘Brutor’ in response to *Plasmodiophora brassicae* pathotype 5X. The annotation of genes that demonstrated opposite expression between the genotypes is shown. LA, Laurentian; BR, Brutor.

At 21 dai, nine genes were upregulated in the susceptible host and downregulated in the resistant one (**Figure 10**). One of these genes, protein phosphatase 2A (*PP2A*), is a key regulator of root polar auxin transport (Rashotte et al., 2007) and was implicated in brassinosteroid regulation upon *P. brassicae* infection (Schuller et al., 2014). This gene is an important candidate susceptibility factor, given the relationship between auxin regulation and clubroot development. Another gene upregulated in ‘Brutor’ and downregulated in ‘Laurentian’ at 21 dai was a tonoplast intrinsic protein 2 (*TIP2*). This gene was shown to interact specifically with an effector from a root knot nematode (Xue et al., 2013), suggesting an additional similarity between nematode-host and *P. brassicae*-host interactions.

Some of the transcripts upregulated in ‘Laurentian’ and downregulated in ‘Brutor’ 21 dai (**Figure 10**) were discussed previously (e.g., *SARD1*), while the remaining genes indicated alterations in development, resource allocation and potential hijacking of the host molecular machinery. These included: i) a NAC-containing protein 83 (*NAC083*), also known as VND-interacting 2, which interacts with the VASCULAR-RELATED NAC-DOMAIN7 (*VND7*) to negatively regulate xylem formation (Yamaguchi et al., 2010); ii) a transport protein particle (*TRAPPC10*) that acts as a tethering factor to join vesicles that are placed on the cell plate during cytokinesis (Jaber et al., 2010); iii) a nitrate transporter (*NPF7.2* also known as *NRT1.8*) that coordinates nitrogen allocation to the xylem (Zhang et al., 2014); and iv) a myo-inositol oxygenase (*MIOX4*), which reduces myo-inositol levels and thus galactinol levels that can activate defenses against the nematode *H. schachtii* (Siddique et al., 2014). The upregulation of these genes at 21 dai suggests that some of the defense processes in the moderately resistant host may have started to abate at the later stages of infection.

Collectively, our results indicate candidate genes that can be tested for validation of their potential function in resistance, and putative susceptibility factors that may increase resistance if mutated. Future candidate genes in studies comparing other genotypes with other pathotypes may be intersected with the genes found in the current study, to identify core elements of defense against clubroot. The common regulation of these genes will shed light on the basic or constitutive defense mechanisms used by most hosts against *P. brassicae*.

### Shared Genes Between Cultivars at Different Time Points

Since we observed that the moderately resistant genotype presented delayed symptoms based on our phenotypical data, we performed comparisons of the number of genes shared between consecutive time-points between the two genotypes, to contrast these numbers with the common genes at the same time-point. For comparisons where the number of genes in consecutive time-points between cultivars was higher than the number of genes shared in the same time-point, functional enrichment analysis of Biological Process ‘level 3’ gene ontology categories was performed for shared genes in the same time-point and for shared genes in consecutive time-points. After eliminating common functional categories between these two analyses, the unique functional categories corresponding to shared genes in consecutive time-points were obtained.

The number of shared genes, when comparing upregulated or downregulated transcripts between the two genotypes at 7 dai, was always greater than the number of shared genes from either cultivar at 7 dai when compared with the other cultivar at 14 dai (**Supplementary Table S5**). The number of shared upregulated genes at 14 dai was 205, which was higher than the number of shared upregulated genes between ‘Laurentian’ at 14 dai and ‘Brutor’ at 21dai, but not higher than the number of shared upregulated genes between ‘Brutor’ at 14 dai and ‘Laurentian’ at 21 dai (247 genes). The functional enrichment analyses showed that most of the unique categories from genes in common between ‘Brutor’ at 14 dai and ‘Laurentian’ at 21 dai corresponded to nematode-related responses, cell growth and development and cell wall metabolism (**Supplementary Table S5**). This further supports similarities between the processes involved in nematode infection and clubroot development. In addition, the categories corresponding to cell growth and cell wall metabolism confirm that these processes are taking place earlier in ‘Brutor’, consistent with our phenotypic and histological observations. Finally, the number of shared downregulated genes between the two genotypes at 14 dai was smaller than the number of shared downregulated genes between ‘Laurentian’ at 14 dai and ‘Brutor’ at 21 dai, or ‘Brutor’ at 14 dai and ‘Laurentian’ at 21 dai (**Supplementary Table S5**). Unique functional categories being downregulated earlier in ‘Laurentian’ than in ‘Brutor’ indicate potential downregulation of carbohydrate metabolism, which usually allows better allocation of resources to defense mechanisms. In contrast, ‘Brutor’ seemed to turn off genes related to defense mechanisms (e.g., ROS metabolic process, defense response to other organism), earlier than ‘Laurentian’.

These analyses show how the resistant genotype is still affected by *P. brassicae* infection, but its molecular defense mechanisms may play a role in delaying symptom development.

## Data Availability Statement

The datasets generated for this study can be found in the NCBI Sequence Read Archive (SRA) accession number PRJNA597078.

## Author Contributions

LG-G designed and conducted all the experiments, performed all analyses and wrote the manuscript. VM assisted with experiments and provided expertise with respect to the inoculations and greenhouse material. S-FH helped secure project funding and contributed to the original experimental context. SS provided project guidance and edited several versions of the manuscript.

## Funding

The authors thank the Canola Council of Canada and Agriculture and Agri-Food Canada for support provided *via* the Growing Forward 2 and Canadian Agricultural Partnership programs. Funding from Alberta Canola and in-kind support by the University of Alberta is also gratefully acknowledged.

## Conflict of Interest

The authors declare that the research was conducted in the absence of any commercial or financial relationships that could be construed as a potential conflict of interest.
