# Supplementary figures and images for: Response of Brassica napus to Plasmodiophora brassicae Involves Salicylic Acid-Mediated Immunity: An RNA-Seq-Based Study
Source: Front Plant Sci. 2020 Jul 9;11:1025. doi: 10.3389/fpls.2020.01025 (PMC7367028; doi:10.3389/fpls.2020.01025)

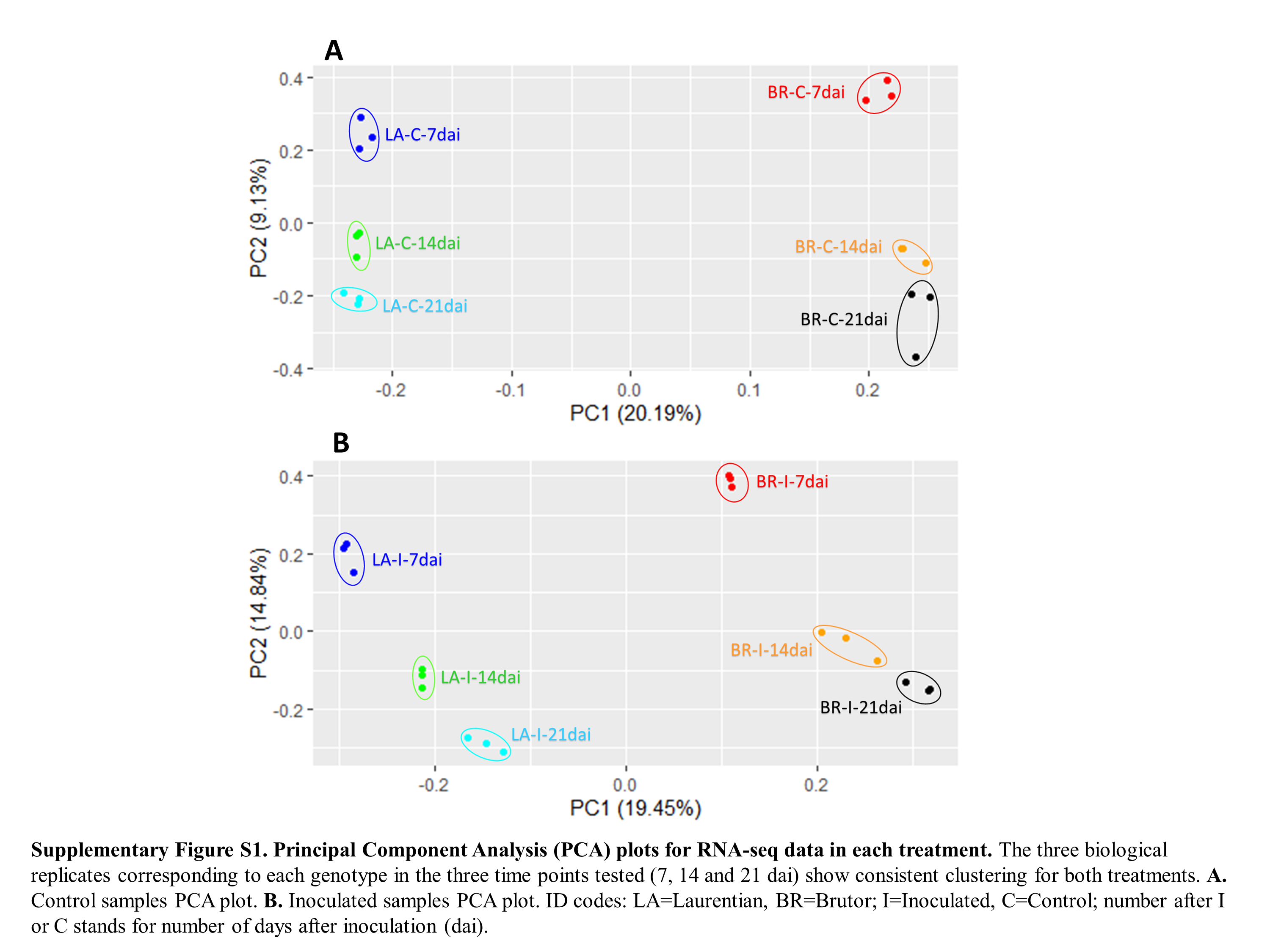

Supplement: Supplementary file 1 [file Image_1.tif]

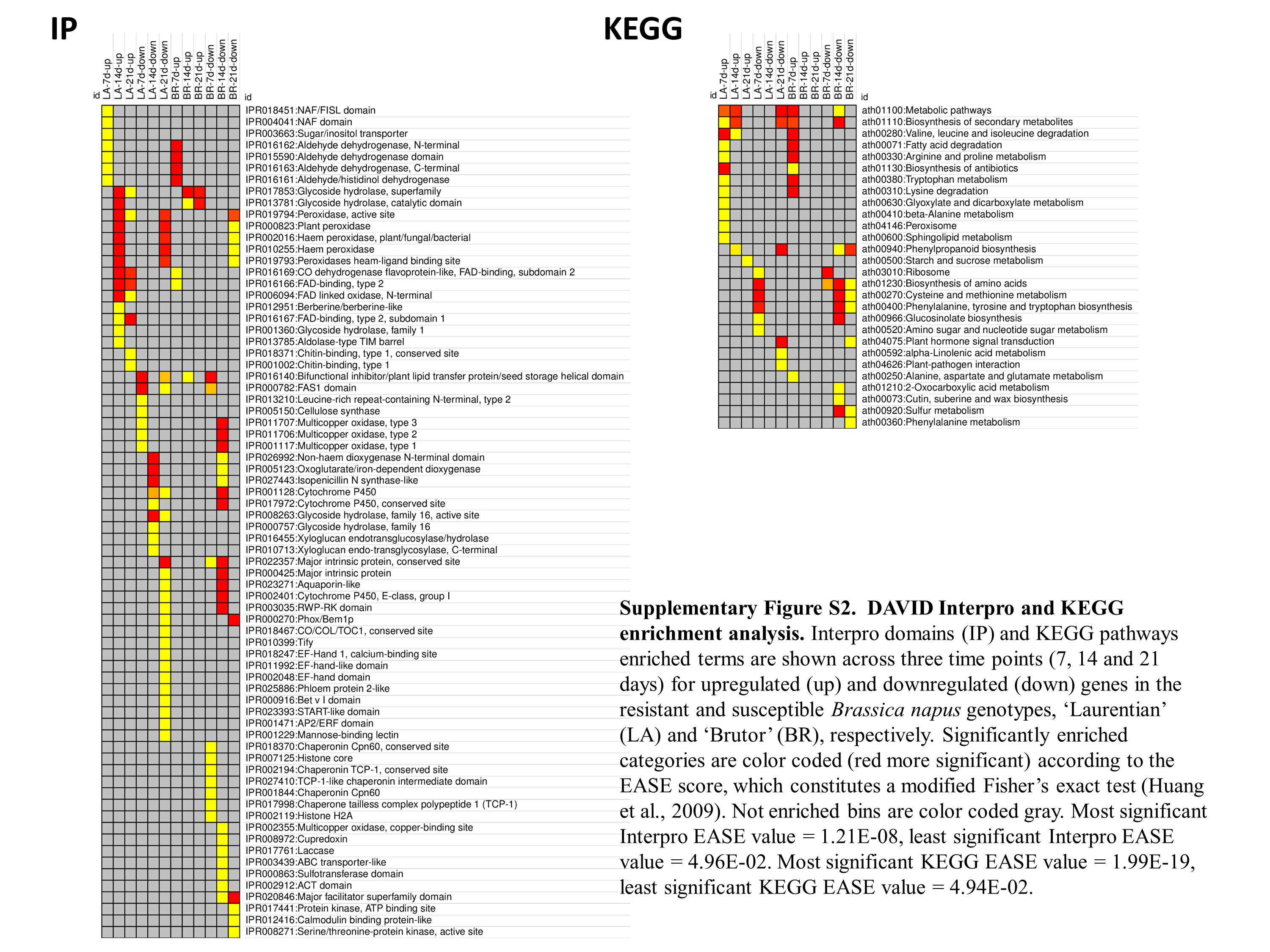

Supplement: Supplementary file 2 [file Image_2.tif]

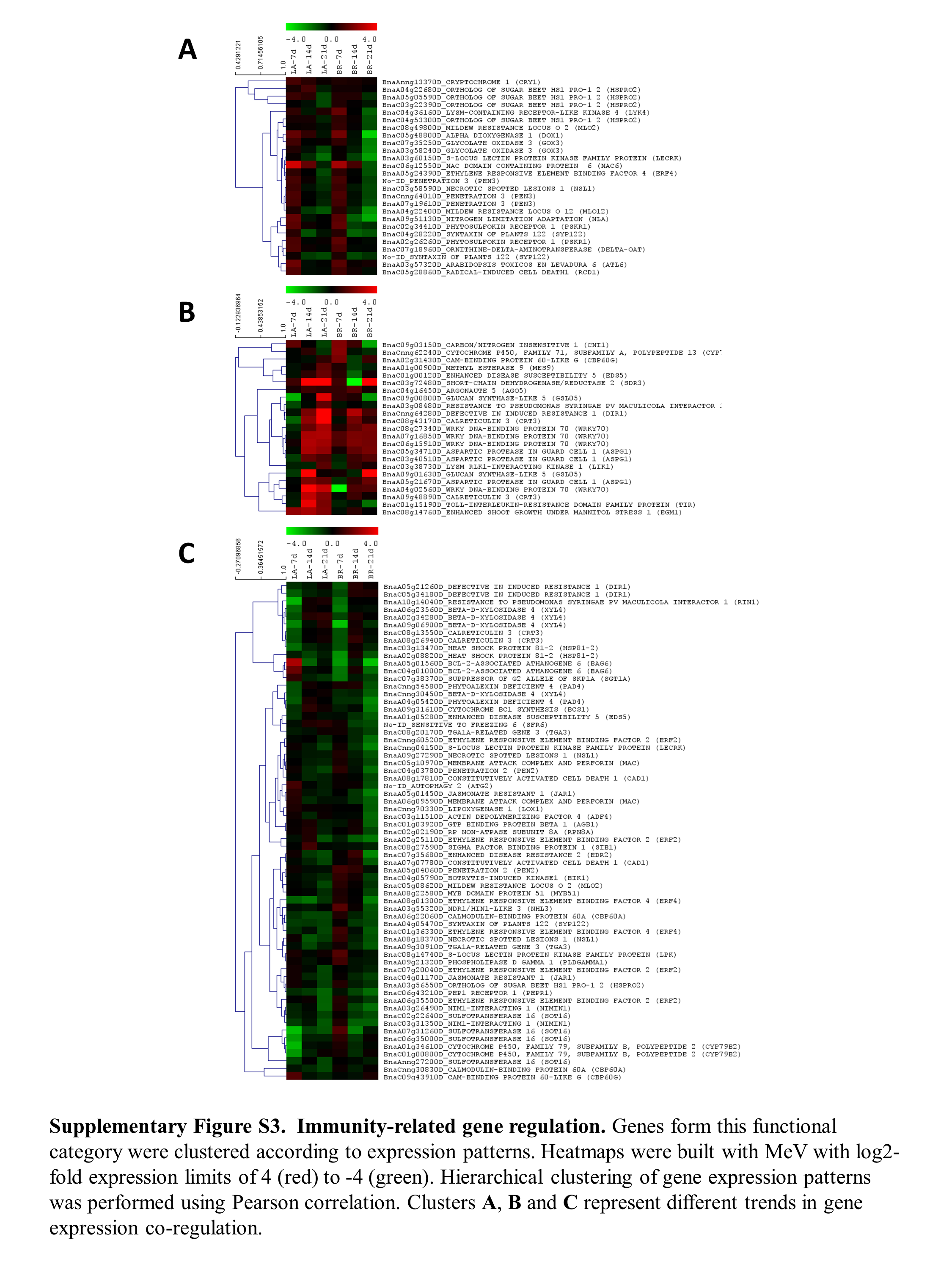

Supplement: Supplementary file 3 [file Image_3.tif]

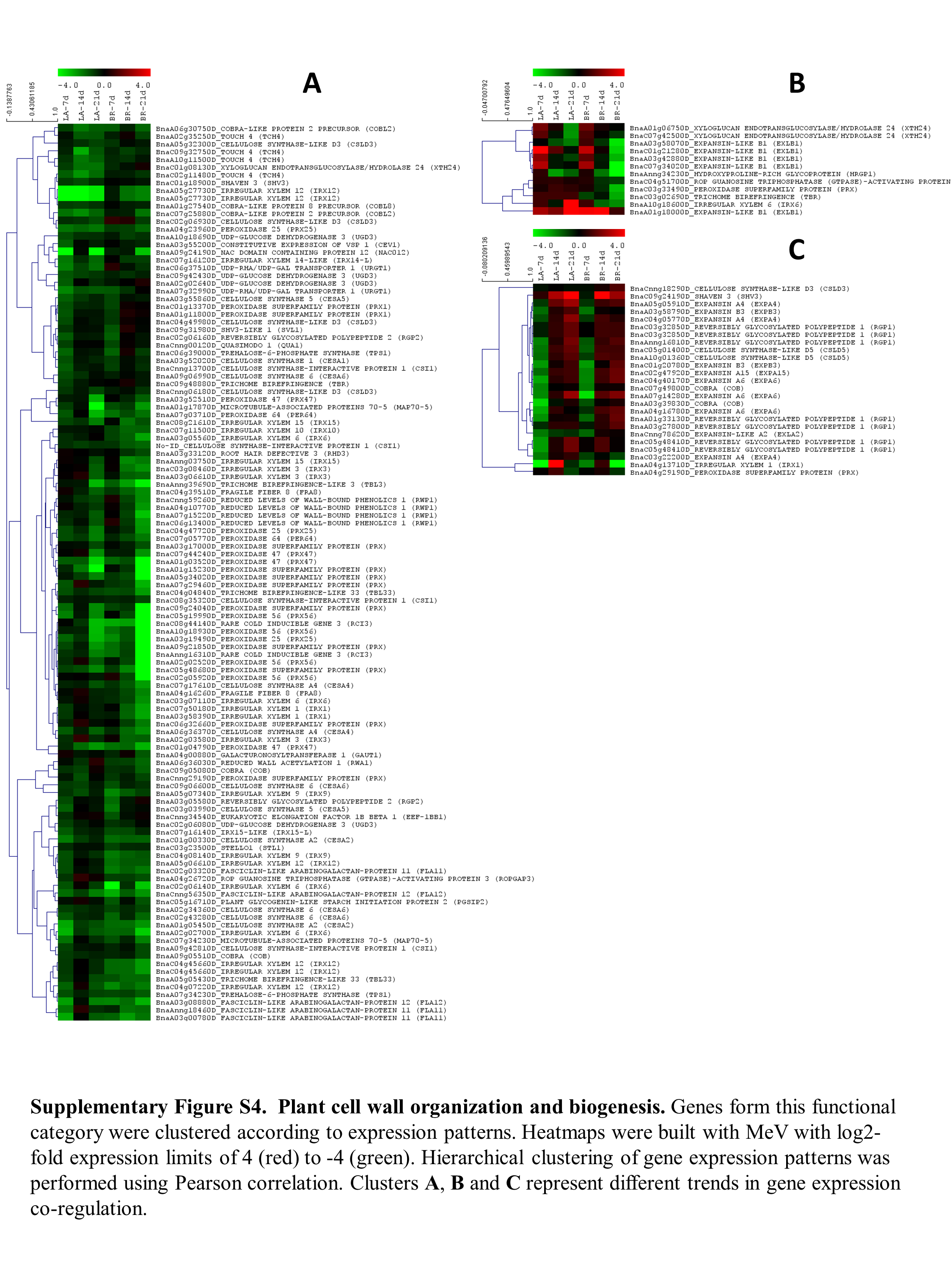

Supplement: Supplementary file 4 [file Image_4.tif]
